# Bilateral Total Hip Replacement for Slipped Capital Femoral Epiphysis in a Young Adult After Growth Hormone Therapy: A Case Report

**DOI:** 10.7759/cureus.102773

**Published:** 2026-02-01

**Authors:** Zelimir Jovanovic, Danilo Jeremic, Lazar Miceta, Nikola D Zarkovic, Nemanja Slavkovic

**Affiliations:** 1 Medicine, University of Banja Luka, Banja Luka, BIH; 2 Orthopaedics and Traumatology, Institute for Orthopaedics Banjica, Belgrade, SRB; 3 Department of Surgery, Faculty of Medicine, University of Belgrade, Belgrade, SRB; 4 Statistics, Faculty of Medicine, University of Belgrade, Belgrade, SRB

**Keywords:** craniopharyngioma, panhypopituitarism, recombinant growth hormone therapy, slipped capital femoral epiphysis, total hip replacement

## Abstract

We present the case of a 26-year-old male who developed bilateral slipped capital femoral epiphysis (SCFE) as a complication of recombinant human growth hormone (rhGH) therapy and was treated with a two-stage bilateral total hip replacement (THR). Growth hormone deficiency was due to a craniopharyngioma, for which curative neurosurgical excision led to panhypopituitarism requiring lifelong hormone replacement and continuation of rhGH therapy. The patient reported bilateral groin pain for two years before presentation; symptoms were initially attributed to short stature and spinal issues, resulting in delayed diagnosis. On presentation, there were clear signs of severe bilateral SCFE, confirmed by radiographs and CT. After shared decision-making, the patient underwent staged bilateral THR, beginning with the left hip. The case illustrates that, with advances in surgical technique and prosthetic materials, THR can be a viable option for young adults with severe SCFE, even when bilateral. It underscores the need for vigilance in monitoring patients on rhGH, particularly those with hypopituitarism, for SCFE, and highlights the importance of multidisciplinary care across endocrinology, neurosurgery, and orthopedic management.

## Introduction

Slipped capital femoral epiphysis (SCFE) is a common pediatric hip disease. In addition to idiopathic SCFE, cases have been reported following recombinant human growth hormone (rhGH) therapy [1]. Among all children treated with growth hormone for various indications, SCFE may account for up to 25.4% of reported adverse events associated with this therapy [2]. While the pathogenetic mechanism is not fully understood, rhGH may decrease growth-plate stability and delay its closure, with factors such as increased weight, high activity, or trauma potentially triggering slippage [3].

SCFE symptoms are often nonspecific, with patients primarily presenting with limp and groin or medial thigh pain, which can mimic other pediatric hip conditions such as Legg-Calvé-Perthes disease or transient synovitis, making prompt diagnosis crucial [4]. Patients with untreated SCFE are at risk for serious complications, including avascular necrosis (AVN) and femoroacetabular impingement (FAI) with secondary degenerative joint disease, underscoring the need for rapid diagnosis. Treatment depends on skeletal age and disease stage [5]. Widely used surgical options include percutaneous fixation with cannulated screws and proximal femoral osteotomies (modified Dunn or Southwick osteotomies) [6]. In some cases, total hip replacement (THR) is warranted, especially in adults who require rapid restoration of hip function [7]. This case report describes SCFE after rhGH treatment in an adult with growth delay due to craniopharyngioma, managed with total hip arthroplasty.

## Case presentation

A 26-year-old male presented to our institute with complaints of left groin and knee pain for over two years, along with right groin and knee pain for one year. He also reported difficulty walking and a progressive decline in his ability to perform daily activities. There had been no known traumatic events preceding the onset of these symptoms.

**Figure 1:**
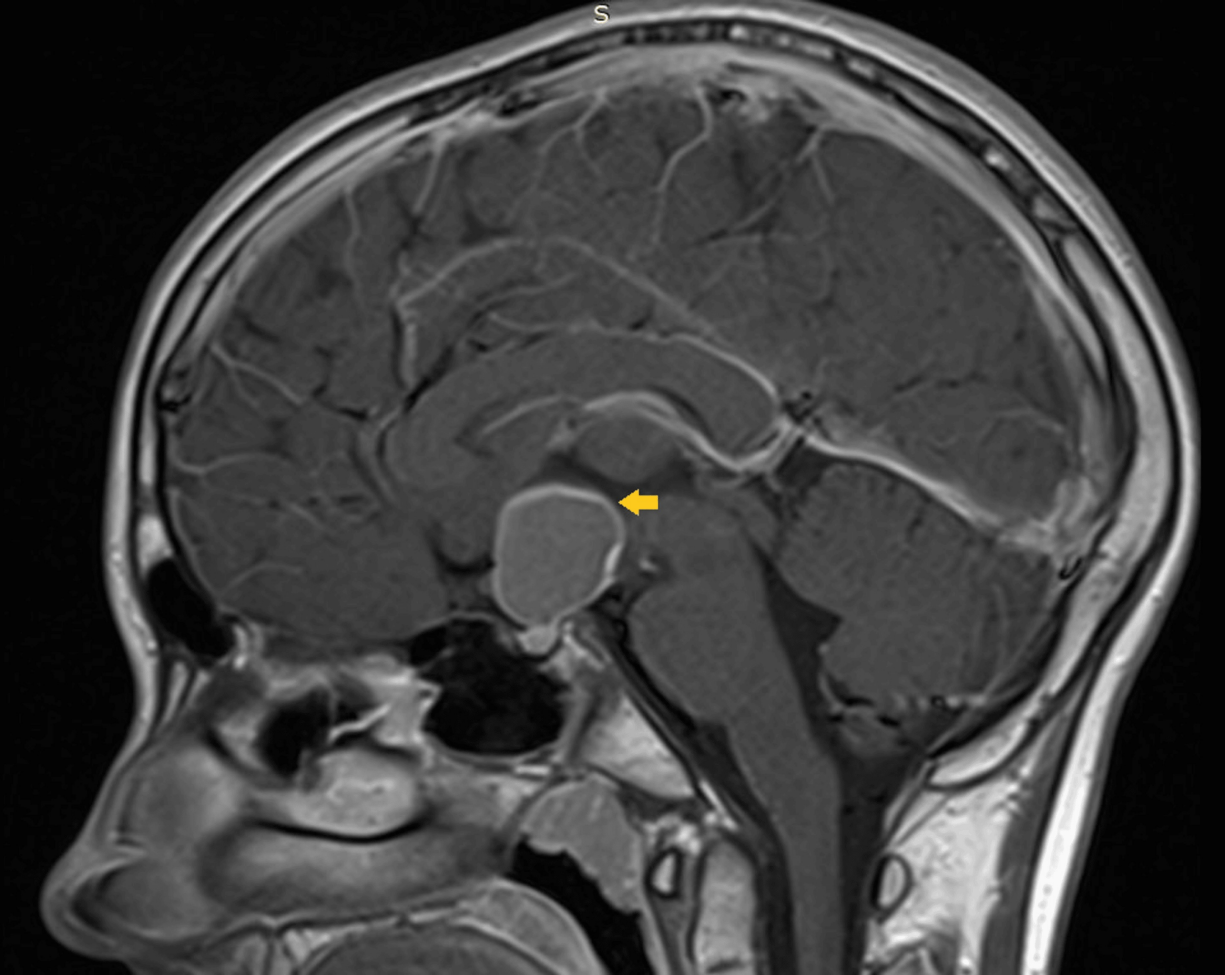
T1-weighted contrast-enhanced MRI of the sellar and suprasellar region demonstrating craniopharyngioma (orange arrow) MRI: magnetic resonance imaging

Anamnesis indicated that the patient had experienced growth delay, first noted at the age of 15, and was initially diagnosed with idiopathic short stature (ISS). He had subsequently begun rhGH therapy, following the standard dosing regimen. Shortly after starting the hormone therapy, the patient had reported experiencing headaches and a decrease in the size of his visual field. These symptoms had prompted an MRI scan of the brain, revealing an expansive tumor in the pituitary fossa (Figure 1). The patient had then undergone neurosurgical treatment, where the pituitary tumor had been excised via a transsphenoidal approach with clean resection margins. Pathohistological analysis had confirmed the diagnosis of craniopharyngioma.

Postoperative iatrogenic panhypopituitarism had been managed with hormone replacement therapy, including thyroxine, cortisone, testosterone, and aldosterone, while rhGH was continued. As a result of the endocrinological treatment, the patient had reached an expected body height above the 95th percentile and achieved nearly complete sexual development, reaching Tanner's stage 4. During adolescence, he had actively participated in sports, especially tennis and basketball. His longitudinal skeletal growth had been completed by the age of 23 years.

During the examination, the patient was conscious, oriented, and able to communicate normally. It was clinically observed that the left leg was shortened by 5 mm and positioned in external rotation of 20 degrees. Tenderness was noted upon palpation of the left groin, along with reduced range of motion (ROM) on both sides, more severely on the left, indicated by a positive Drehmann's sign (flexion possible only when the hip is abducted).

The left hip ROM was recorded as follows: extension/flexion from 0 to 30°, adduction/abduction from 10 to 20°, and internal/external rotation from 5 to 25°. In comparison, the right hip ROM was recorded as follows: extension/flexion from 10 to 70°, adduction/abduction from 15 to 30°, and internal/external rotation from 10 to 30° [8]. The patient's gait was antalgic, characterized by a shortened stance phase on the left leg and an altered angle of progression for the left foot and knee during the swing phase due to external hip rotation. Harris’s hip score (HHS) reflected moderate limitations, with scores of 62 for the left side and 78 for the right side [9].

**Figure 2:**
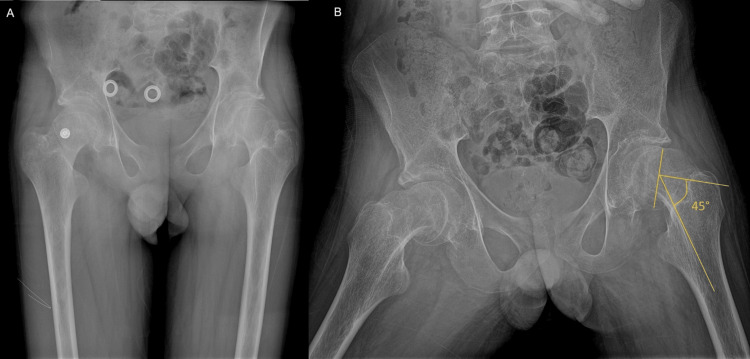
Anteroposterior pelvis view (A) and Lowenstein (frog-leg) view (B) of both hips demonstrating abnormal values of Southwick angle

**Figure 3:**
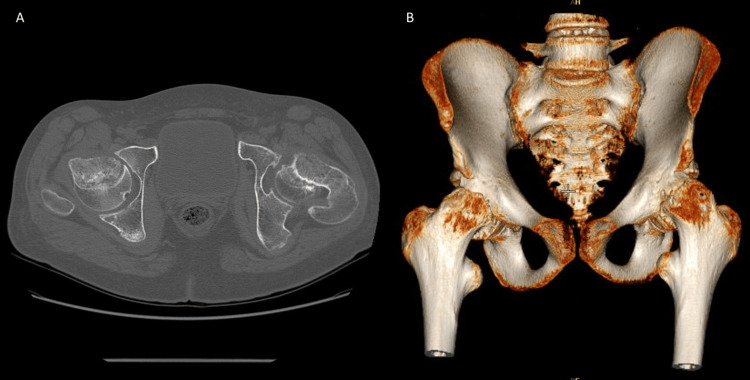
Axial view (A) and 3D reconstruction (B) of CT scan CT: computed tomography

Radiological evaluation included standard X-rays in anterior-posterior (AP) and Lowenstein projections, as well as a CT scan. These tests revealed a grade III SCFE (slippage greater than 50% of the growth plate width), with widening of the growth plates, a positive Trethowan’s sign (Klein's line does not intersect the lateral part of the superior femoral epiphysis), and pathological values of the Southwick angle (Figures 2, 3).

Considering the patient's age, the stage of the disease, the uncertain outcomes of alternative surgical methods, and the patient's request for rapid functional recovery, the decision was made to perform a two-stage THR, starting with the left hip. Before the surgery, the patient received a single dose of antibiotics (first-generation cephalosporins). Preoperative surgical templating was carried out using the TraumaCad® program (BrainLAB, Munich, Germany).

**Figure 4:**
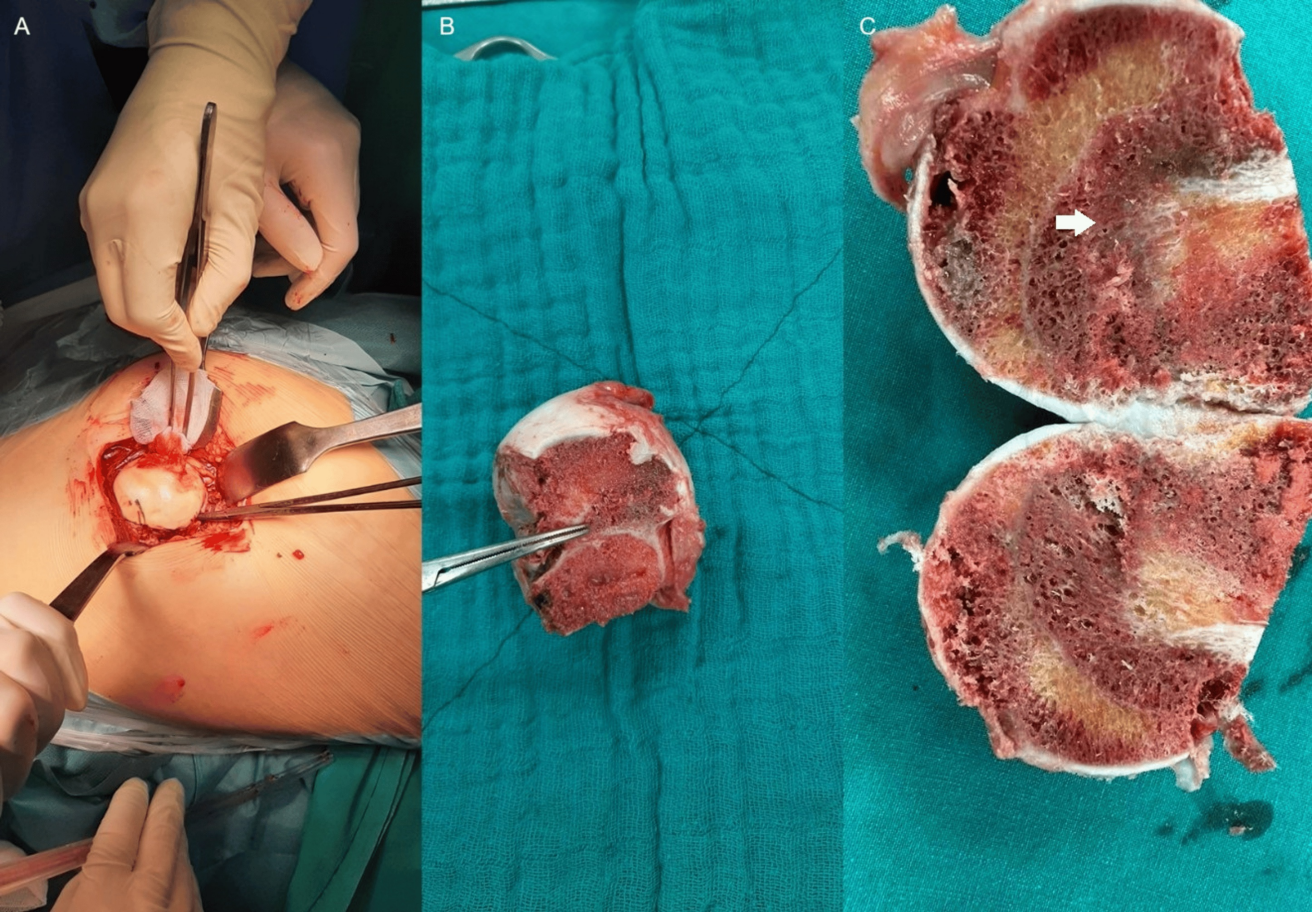
Intraoperative finding of slipped capital femoral epiphysis Surgical dislocation of the hip (A); extracted femoral head and neck specimen (B and C) showing epiphyseal slip (white arrow)

A left THR was performed using a posterolateral (Southern-Moore) approach. During the procedure, we observed a severe degree of slippage of the left femoral head, which resulted in a loss of the normal anatomical features at the epiphyseal-metaphyseal transition (Figure 4).

We chose to use an uncemented THR with a ceramic-on-ceramic bearing, consisting of a Pinnacle® acetabular shell, a ceramic liner and head, and a Corail® uncemented stem (De Puy, Synthes, Warsaw, IN). Six months later, the same procedure was performed on the right hip.

**Figure 5:**
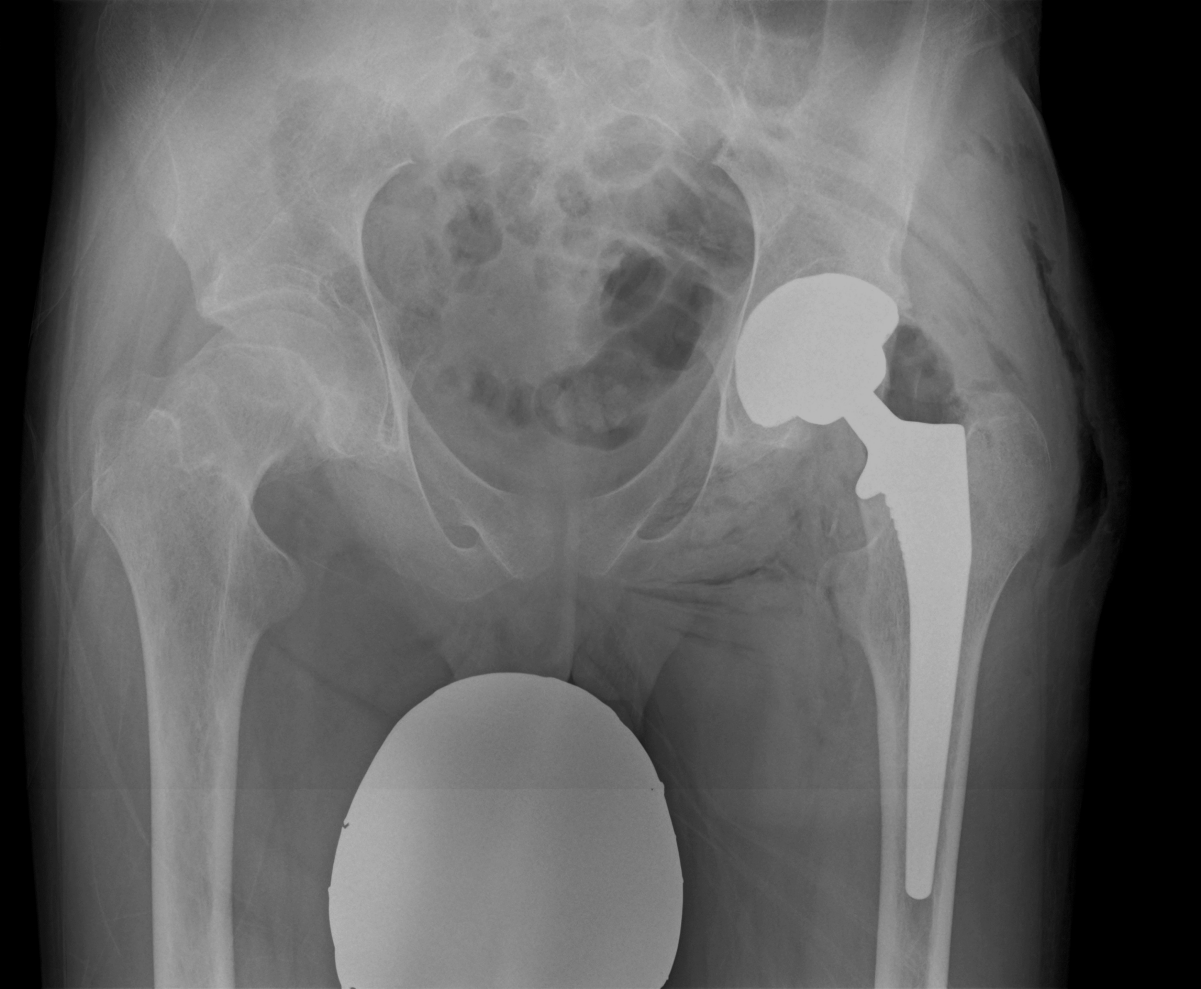
X-ray obtained on regular control after left hip THR THR: total hip replacement

**Figure 6:**
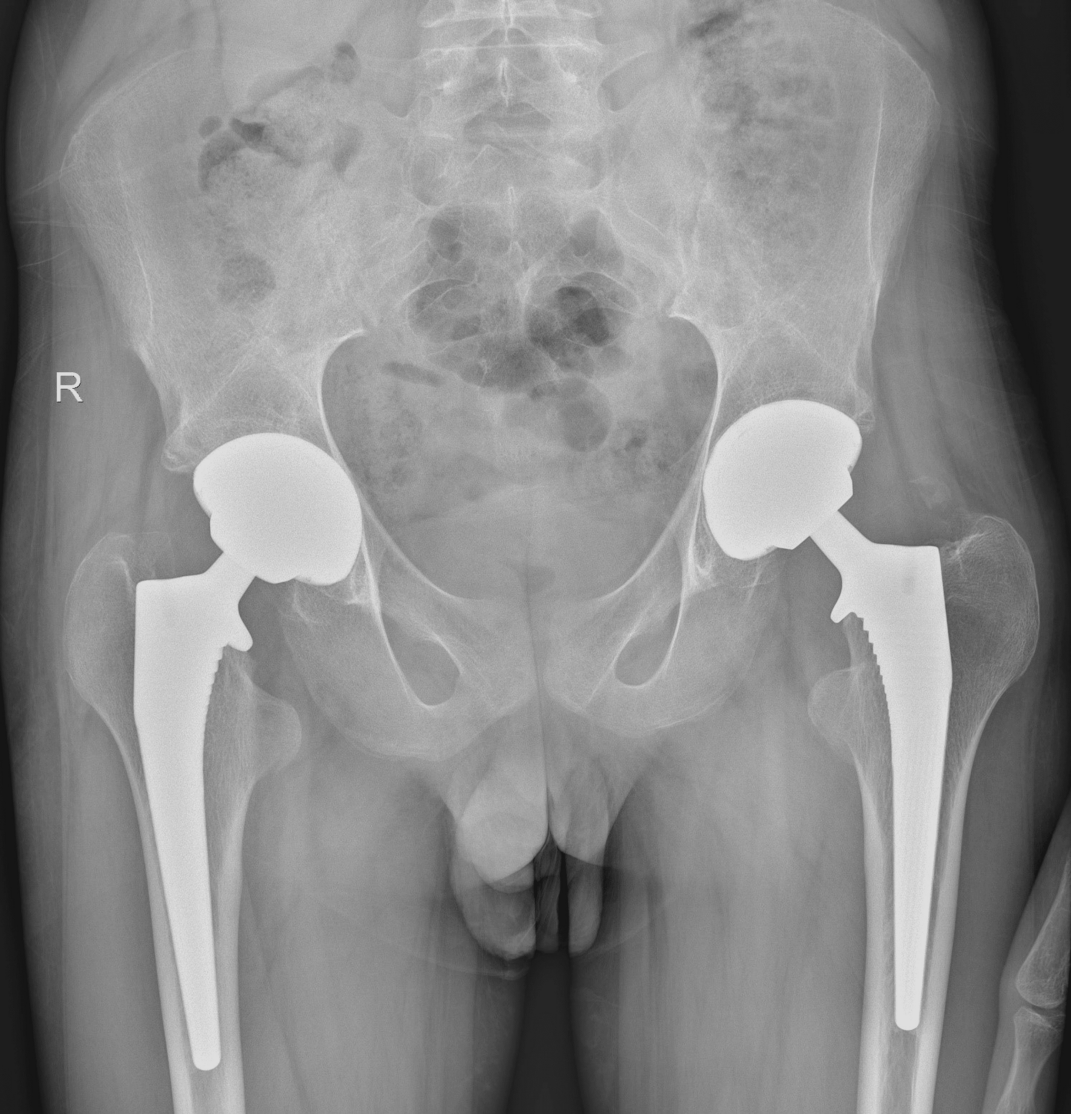
Pelvis X-ray after a follow-up period of two years There were no signs of implant loosening

The standard rehabilitation protocol commenced on the first postoperative day, followed by an additional three-week period of inpatient recovery at a specialized rehabilitation center after both surgeries. Regular follow-up appointments were conducted in accordance with the established protocols, with the final visit occurring two years after the first surgery and one and a half years after the second. The leg length discrepancy was corrected, and the patient's gait and ROM improved in both hips. The improvements in the left hip were as follows: extension/flexion from 15 to 110°, adduction/abduction from 20 to 45°, and internal/external rotation from 30 to 40°. For the right hip, extension/flexion averaged from 20 to 120°, adduction/abduction from 30 to 50°, and internal/external rotation from 35 to 50° [8]. At the last follow-up, HHS for the left hip was 95, while the right hip had a score of 93 [9]. Radiological examinations confirmed that the components were well-seated, with no signs of complications (Figures 5, 6).

## Discussion

SCFE is the most common hip joint condition in adolescents, with an incidence of 8 to 13 cases per 100,000 children aged 10-16 years [1-5]. The causes of SCFE involve complex interactions between local mechanical factors and systemic endocrine influences. This condition can result from increased stress on a normal growth plate, often due to obesity or vigorous physical activity, or from physiological forces acting on a growth plate compromised by systemic conditions, such as endocrine disorders [5-7]. Various hormones, including growth hormone, thyroid hormones, and sex hormones, play a role in the maturation of the growth plate [10]. Growth hormone can potentially delay chondrocyte differentiation and increase the diameter of the growth plate, which in turn affects its stability [11].

Endocrine disorders should be suspected in cases that fall outside the typical age range (younger than 10 or older than 16), particularly if there are additional symptoms indicating hormonal issues [7]. Bilateral involvement of SCFE is also a sign of systemic factors, as idiopathic cases involve both hips in only 10-20% of cases, compared to higher rates in individuals with endocrine comorbidities. Metachronous slips - where the condition develops in the opposite hip at a later point in time - can occur 12-18 months after the initial diagnosis [4].

The case presented highlights how biomechanical and hormonal factors influence the onset and progression of SCFE [12]. In our patient, the administration of rhGH due to iatrogenic panhypopituitarism was likely the cause of SCFE. The contribution of increased physical load cannot be disputed because our patient had actively participated in sports for a long period while on rhGH therapy. However, symptoms only emerged after the patient had stopped playing sports. Unusually, these symptoms had appeared in early adulthood, which is outside the typical age range for SCFE. This may be linked to inadequate response to testosterone therapy, which could have delayed the closure of the growth plates [7]. This is supported by signs of incomplete secondary sexual characteristics, reduced muscle mass, and decreased bone density.

When determining the therapeutic approach for this case of chronic, stable SCFE, several factors were considered. One possible treatment option was a Southwick intertrochanteric osteotomy. The objective of this biplanar osteotomy is to bring the segment distal to the osteotomy site into a position of valgus and flexion [5]. This correction addresses the deformity resulting from SCFE and enhances ROM. Additionally, by restoring normal anatomical relationships in the proximal femur, this procedure helps ensure the proper distribution of loads and joint reaction forces in the hip, which may prevent or slow the progression of secondary osteoarthritis [7]. Osteoarthritis can occur as part of the natural progression of both untreated and treated SCFE, often due to FAI [13]. Individuals with SCFE are likely to develop degenerative hip disease if they live long enough, and complications from surgical treatment, such as AVN and chondrolysis, can markedly accelerate this progression.

Besides the Southwick procedure, other proximal femoral osteotomies can also serve as valid treatment options for chronic, stable cases of SCFE. Imhauser described an intertrochanteric osteotomy that is slightly more proximal than the Southwick and aims to correct the deformity in all three planes by creating a position of flexion, valgus, and internal rotation [14]. This procedure may achieve greater correction and better functional outcomes compared with the Southwick osteotomy, especially when combined with osteochondroplasty to address issues of metaphyseal remodeling and FAI [15]. Kramer’s base-of-the-neck osteotomy is another extracapsular option, as are the intracapsular modified Dunn and Fish procedures. These intracapsular osteotomies are performed through a surgical hip dislocation approach and aim to remove the posterior metaphyseal callus and shorten and valgize the femoral neck. However, they are associated with significantly higher rates of complications, such as AVN and chondrolysis, compared with extracapsular osteotomies, because the surgical approach may compromise the delicate vascularization of the femoral head [16].

While many studies report satisfactory outcomes following Southwick and other proximal femoral osteotomies, these procedures are associated with several significant challenges. Firstly, they are technically complex and should ideally be performed in specialized university centers that manage a sufficient number of patients requiring this type of surgery. Additionally, numerous potential complications may arise during the immediate and early postoperative period, including AVN and chondrolysis. AVN is a rare complication following Southwick osteotomy, which is one advantage of this procedure over intracapsular osteotomies such as the modified Dunn and Fish procedures [15]. However, chondrolysis is a well-documented complication of Southwick osteotomy. A study by El-Mowafi et al. reported a statistically significantly higher incidence of chondrolysis in patients undergoing the Southwick procedure compared with those treated with the Kramer base-of-the-neck osteotomy, with chondrolysis seen in 25% of cases [16].

In addition to these complications, lower limb length discrepancies after intertrochanteric osteotomy may pose significant challenges, as the operated leg can end up shorter or longer than the non-operated leg. Studies show that this leg length difference can range from 0.5 to 3 cm, and in some cases, the discrepancy was substantial enough to disrupt the normal biomechanics and balance of the spinopelvic segment, sometimes necessitating a corrective osteotomy on the opposite leg to achieve leg length equality [14]. This issue can be particularly troublesome in patients with bilateral SCFE. Furthermore, the rehabilitation process following proximal femur osteotomy is lengthy and complex, often involving several months of restricted weight-bearing on the operated leg. This limitation can be especially challenging for adult patients who anticipate a swift return to normal hip function post-surgery.

Short-term studies evaluating outcomes after Southwick osteotomy have reported excellent results, showcasing significant increases in range of motion and improvements in functional scores [17]. However, long-term studies tracking patients over 10 to 20 years post-surgery revealed that many adolescents and young adults who underwent osteotomy developed both clinical and radiological signs of degenerative hip disease. Ultimately, when non-operative treatment options are exhausted, they may require THR [7].

Performing THR after a proximal femur osteotomy is highly complex. The frequency of complications can approach that seen in revision THR cases [18]. Various studies indicate that the duration of the operation is prolonged, resulting in increased blood loss. The altered anatomy of the proximal femur and the presence of sclerosis at the osteotomy site complicate the preparation of the femoral canal, making it challenging to achieve the appropriate anteversion angle of the stem and ensure the proper size for initial stability. This also increases the risk of iatrogenic intraoperative femoral fractures [19]. In addition to this heightened risk of intraoperative complications and an extended rehabilitation period, some studies have indicated that prior femoral osteotomies can compromise the long-term survival of the femoral component of the prosthesis, leading to a higher incidence of aseptic loosening of the femoral stem [20]. Some authors have suggested the use of custom-made femoral implants [21].

Given these considerations, the decision was made to perform THR. Due to the patient's age, ceramic bearing surfaces were used, as they show superior friction coefficients and wear properties compared with standard metal-on-polyethylene pairs. This therapeutic approach is not typically standard for this age group, and to the best of the author's knowledge, there are few recorded cases of SCFE treated with THR. While THR allows rapid recovery and demonstrates excellent functional results, it is associated with several long-term complications, including aseptic loosening of the components, wear, chronic periprosthetic joint infection, dislocation, and periprosthetic fractures [22]. However, considering improvements in the tribological profile of prosthetic materials and advancements in surgical techniques, which both contribute to a longer lifespan of the prosthetic system, we believe that THR will have an increasingly important role in the treatment of SCFE.

## Conclusions

SCFE is a condition with a complex range of causes, involving multiple local and systemic predisposing factors. While diagnostic algorithms have been developed to identify specific clinical and radiological signs that aid in diagnosis, there remains ongoing debate within the scientific community regarding the most effective treatment approaches. It is essential to conduct further studies to evaluate the long-term outcomes of different treatment options. Additionally, we recommend that routine follow-up appointments at orthopedic clinics become standard practice for all patients receiving rhGH therapy. The introduction of new and more biocompatible materials for THR is expected to enhance treatment strategies and improve outcomes for patients with SCFE.
